# No impact of a prescription booklet on medication consumption in nursing home residents from 2011 to 2014: a controlled before–after study

**DOI:** 10.1007/s40520-020-01670-5

**Published:** 2020-08-03

**Authors:** Stéphane Sanchez, Cécile Payet, Marie Herr, Fiona Ecarnot, Caroline Blochet, Didier Armaingaud, Jan Chrusciel, Jean-Luc Novella, Rachid Mahmoudi

**Affiliations:** 1 Fondation Korian Pour le Bien Vieillir, 25 Rue Balzac, 75008 Paris, France; 2 Centre Hospitalier de Troyes, Pôle Information Médicale Évaluation Performance, 101 Av Anatole France, 10000 Troyes, France; 3 Hospices Civils de Lyon, Pôle Information Médicale Evaluation Recherche, 162 Av Lacassagne, 69003 Lyon, France; 4 Université de Versailles Saint Quentin, UMR 1168 Vieillissement et Maladies chroniques, Approches épidémiologique et de santé publique, 16 Av Paul Vaillant Couturier, 94807 Villejuif, France; 5 Department of Cardiology, University Hospital Jean Minjoz, Boulevard Fleming, 25000 Besançon, France; 6 EA3920, University of Burgundy Franche-Comté, 19 Rue Ambroise Paré, 25000 Besançon, France; 7 Medissimo, 8 Rue Charles Edouard Jeanneret, 78300 Poissy, France; 8 Department of Geriatrics and Internal Medicine, Maison Blanche Hospital, Reims University Hospitals, 45 Rue Cognacq-Jay, 51000 Reims, France; 9 Faculty of Medicine, University of Reims Champagne-Ardenne, 51 Rue Cognacq-Jay, 51100 Reims, France

**Keywords:** Polypharmacy, Nursing homes, Geriatrics, Health policy

## Abstract

**Background:**

Older persons are particularly exposed to adverse events from medication. Among the various strategies to reduce polypharmacy, educational approaches have shown promising results. We aimed to evaluate the impact on medication consumption, of a booklet designed to aid physicians with prescriptions for elderly nursing home residents.

**Methods:**

Among 519 nursing homes using an electronic pill dispenser, we recorded the daily number of times that a drug was administered for each resident, over a period of 4 years. The intervention group comprised 113 nursing homes belonging to a for-profit geriatric care provider that implemented a booklet delivered to prescribers and pharmacists and specifically designed to aid with prescriptions for elderly nursing home residents. The remaining 406 nursing homes where no such booklet was introduced comprised the control group. Data were derived from electronic pill dispensers. The effect of the intervention on medication consumption was assessed with multilevel regression models, adjusted for nursing home status. The main outcomes were the average daily number of times that a medication was administered and the number of drugs with different presentation identifier codes per resident per month.

**Results:**

96,216 residents from 519 nursing homes were included between 1 January 2011 and 31 December 2014. The intervention group and the control group both decreased their average daily use of medication (− 0.05 and − 0.06). The booklet did not have a statistically significant effect (exponentiated difference-in-differences coefficient 1.00, 95% confidence interval 0.99–1.02, *P* = .45).

**Conclusion:**

We observed an overall decrease in medication consumption in both the control and intervention groups. Our analysis did not provide any evidence that this reduction was related to the use of the booklet. Other factors, such as national policy or increased physician awareness, may have contributed to our findings.

**Electronic supplementary material:**

The online version of this article (10.1007/s40520-020-01670-5) contains supplementary material, which is available to authorized users.

## Background

Older persons are particularly exposed to adverse events from medication, due to the physiological changes that occur with normal aging as well as the frequent presence of multiple pathologies [1]. Multi-morbidity increases the risk of exposure to polypharmacy, which augments the risk of drug interactions and adverse effects, which can in turn result in additional prescriptions being issued [1–3]. There are various definitions of polypharmacy, based on the number of prescribed drugs and/or their type and the duration of treatment [4]. Some therapeutic classes (e.g., non-steroidal anti-inflammatory drugs, opioid analgesics, neuroleptics, and antidementia drugs) are more likely than others to be potentially inappropriate in older persons [5, 6]. Numerous studies have reported a high use of medication in nursing homes, with a substantial risk of inappropriate prescriptions [7–9]. Similarly, there is a considerable body of evidence attesting to the fact that the rate of use of potentially inappropriate medications remains high among nursing home residents [10–13].

However, before any interventions can be planned to improve the quality of medical prescriptions and reduce polypharmacy, it is necessary to identify suitable indicators of inappropriate medication use in nursing homes. The most common such indicator is Beers’ list of inappropriate medications, first introduced in 1991 [14], although a number of groups have since developed similar quality indicators, including an update of Beers’ criteria [15–18]. More recently, an expert consensus was published, named the EU(7)-PIM list, covering prescription drugs commercialized in seven European countries [19]. Actions targeting these indicators may decrease the potential for adverse effects, interactions, or medication misadventures. For example, interventions such as drug reviews by doctors or pharmacists have been shown to be effective in reducing the burden of medication in older patients [20–23]. A multifaceted intervention including education, written material, real-time reminders, and outreach visits was shown to be effective in reducing antimicrobial prescriptions for suspected urinary tract infections in residents of nursing homes without a significant impact on hospitalizations or mortality [24]. Other educational approaches (e.g., small group interactive sessions for nurses, videotapes, written material, outreach visits, e-learning modules, and one on one interviews with physicians) were also shown to have a beneficial effect on potentially inappropriate prescriptions [8, 9, 25–29]. Finally, standard instruments such as the Screening Tool of Older Persons’ Prescriptions (STOPP) [30, 31] have also been found to be effective in identifying potentially inappropriate prescribing in older people, although, in a Norwegian study, only 44% of serious adverse drug events in patients acutely admitted to a medical department could have been prevented by STOPP compliance [32].

In this context, the main objective of this study was to compare changes in prescription practices between nursing homes that introduced a booklet designed to aid with prescribing in older nursing home residents, and those where no such booklet was introduced. Our secondary objective was to assess the impact of this intervention on the rates of prescription of frequently prescribed drug classes (antihypertensive drugs and hypnotics).

## Methods

### Study design

#### Development of the booklet

The French Korian group of residential homes for dependent older persons decided to implement a policy of safer drug prescribing. To this end, the Korian group convened a group of experts to develop a booklet aimed at providing support to health professionals for the initiation and reevaluation of medical treatments for older people in nursing homes [available at: https://news.korian.fr/download/123 (Access date: 27 June 2020)]. The ultimate objective was to reduce iatrogenic effects due to non-optimal or improper medication use. The booklet was prepared by national opinion leaders and experts working in the field of geriatrics and pharmacology, and designed to provide an informed perspective based on the latest guidelines and published evidence. It provides an index of standard medication suitable for use in older nursing homes residents, and alternative drugs for use in specific situations. It lists the medications suitable for use, while drugs whose efficacy in older patients is not proven, or whose risk–benefit ratio is unfavorable, are not listed. It is not meant to be a substitute for the Summary of Product Characteristics for each drug. As a policy tool to improve the safety of prescriptions in the elderly, the aim of the booklet was to assist health professionals in complying with geriatric norms when they prescribe. This was a joint endeavor involving doctors treating nursing home residents, pharmacists in charge of managing the in-house pharmacy in each nursing home, and university-based research teams. The booklet presents a summary of institutional guidelines (issued by the French National Authority for Health, the National Agency for Drug Safety), guidelines from various professional societies (e.g., cardiology, rheumatology, etc.), and from the international literature, the drug directory of the National Institute for Health and Care Excellence [33], and from analysis of more than 100,000 drug prescriptions issued in nursing homes.

#### Communication about the booklet

The booklet was distributed to all prescribers and pharmacists in the nursing homes of the Korian group; these nursing homes constitute the Intervention group of the study; all were private for-profit establishments. In support of the dissemination of the booklet, there was also an intensive communication campaign targeting all health professionals involved in medication prescription, administration, or monitoring. A series of presentations were organized in each establishment to cater for variations in staff presence. During these formal presentations, the booklet’s aims, content, and structure (different sections, where to find relevant information…) were presented to all nurses, pharmacists, and prescribers. In France, the practitioners who write the prescriptions for the residents of nursing homes are local general practitioners (GPs) who are generally not full-time employees of the nursing home. In addition, the booklet was made freely available in the staff room.

### Database

To evaluate the effect of this booklet on prescriptions, we retrospectively compared the daily number of times that a drug was administered to each patient between nursing homes of the intervention and control groups using a difference-in-differences design. The data were derived from a database containing information about prescriptions in 519 French nursing homes that were using an electronic pill dispensing system manufactured and marketed by Medissimo (Poissy, France) at the time of the study. This system has previously been described [9]. Briefly, the pill dispenser contains 28 days of treatment, with as many compartments as drugs and times of administration. It is prepared by qualified staff in the pharmacy. During the preparation, information about the individuals (age and sex) and their prescriptions (drugs, dose, time of administration, duration of prescription, and price of each drug) is entered into specific software to ensure traceability, and accurately describes drug consumption [34]*.*

### Main outcome

We compared the average daily number of times a drug dosage unit was administered for each nursing home resident between 1 January 2011 and 31 December 2014 in 113 nursing homes of the Korian group who introduced the booklet (all private, for-profit establishments), versus 406 nursing homes who did not introduce the booklet (private for-profit, private not-for-profit, and public establishments). We chose the number of daily drug dosage units (e.g., a drug from a single therapeutic class administered morning, noon and evening would count as 3 dosage units) as the main outcome, first, because it was reliably recorded in our database, and second, because drug dispensing has been shown to be a suitable proxy for drug consumption [35, 36]. No specific definition of polypharmacy was applied in our study, since polypharmacy (as a binary variable, i.e., more or less than a specific number of drugs) was not recorded. The 4-year study period was chosen to capture long-lasting changes in prescriptions, and to account for individual variations in prescriptions due to changing patients’ needs. Since physicians can change prescriptions more or less immediately, and the maximum authorized duration of any one prescription in France is 3 months, it was assumed that a period of 4 years would be sufficient for any changes to become apparent. The data were anonymized and communicated to the authors by the manufacturers of the electronic pill dispenser. Nursing homes were not randomized, however, they were similar in terms of the number of beds. Trends in drugs consumption in the intervention and control groups of nursing homes were compared over two periods (before and after the implementation of the booklet). The objective of the difference-in-differences approach was to take into account natural trends in prescriptions by including data from a control group in the analysis [37]. The control group was chosen to accurately represent trends in drug prescriptions unrelated to the booklet.

### Secondary outcomes

We also investigated the total number of different drugs prescribed for each resident and the use of antihypertensive drugs and hypnotics (the most prescribed therapeutic classes) within the nursing homes of the intervention group prior to and after introduction of the booklet (May 2012), using the other nursing homes with available data as a control group.

**Fig. 1:**
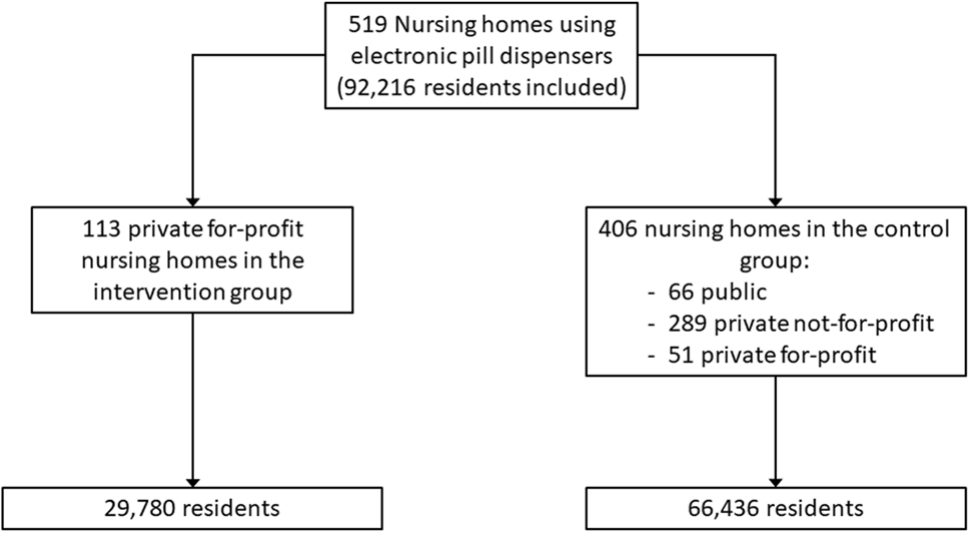
Flowchart of the study

The nursing homes included in the present study are presented in the flowchart (Fig. 1).

### Data collection and variables used to assess medication use

We collected data regarding the characteristics of the residents (e.g., gender, age at admission); characteristics of the nursing homes [e.g., the number of beds, intervention or control group, status (private/public and for-profit/not-for-profit)]; indicators for the use of drugs for chronic diseases with regard to polypharmacy [i.e., the average monthly number of Presentation Identifier Codes (PIC), the average daily number of times when medications were administered (morning, afternoon, evening, and bedtime)] [38],and indicators for the classes that are most often prescribed (i.e., antihypertensives and hypnotics). The different classes of medications were defined based on the World Health Organization Anatomical Therapeutic Chemical classification. We also assessed the total cost of the medications that were consumed over the study period. The value of the dispensed drugs was determined by calculating the sum of the unit price (in Euros) of all boxes of medications delivered during the study period.

### Statistical analysis

The characteristics of the nursing homes and residents are presented by group (i.e., intervention or control group). Categorical variables are reported as number and percentage. Quantitative variables are reported as mean ± standard deviation or as median [range].

Difference-in-differences multilevel Poisson regressions were performed to explain the average number of times drugs were administered per day per resident, taking into account the fact that residents cannot be considered to represent independent observations [39]. The Poisson regression model was chosen to match the data distribution. Explanatory variables were: the group, the period, as well as the group × period interaction (difference-in-differences indicator), to evaluate the effect of the booklet. The practitioners who write the prescriptions for the residents of nursing homes are GPs who are generally not full-time employees of the nursing homes, so the status of the prescribers remains the same for all nursing homes (whether private for-profit, private not-for-profit, or public). Conversely, nurses, administrators, and other staff could differ between the nursing homes, so analyses were adjusted for nursing home status as a random effect to account for this. Analyses were also adjusted for the number of beds in the nursing home, to account for size.

Results are expressed as Relative Risks (RR) with 95% Confidence Intervals (CI). The difference-in-differences estimator was expressed as the ratio of RR, since it is a measure of the change in the RR between the intervention group and the control group. It is interpreted in the same way as the RR. A ratio of RR greater than 1 indicates a higher number of medications in the intervention group than in the control group. Analyses were considered exploratory; therefore, no alpha risk adjustment method was applied. Data management and statistical analyses were performed using SAS software version 9.4 (SAS Institute Inc., Cary, North Carolina, USA).

## Results

### Characteristics and course of drug prescriptions

A total of 519 nursing homes in France were included [66 public (12.7%), 289 private not-for-profit (55.7%), and 164 private for-profit (31.6%)], involving a total of 96,216 residents between 1 January 2011 and 31 December 2014 (Fig. 1). The characteristics of the nursing homes are presented in Table 1. The cost of medication is presented in Supplementary Table S1 (see Additional File 1). As shown in Table 2, there was a significant decrease in the average daily number of drugs administered per resident between the first and second periods in both groups (− 0.05 and − 0.06, for the intervention and control groups, respectively, *p* < 0.001 for after versus before), and in the average daily use of hypnotics (− 0.04 and − 0.05, *p* < 0.001 for each, after vs before). A significant decrease was noted in the average number of PIC used per month in both the control and intervention groups (− 0.26 and − 0.32, *p* < 0.001) over the study period.

**Table 1:** Characteristics of the nursing homes

	Intervention group	Control group
Nursing homes (*n*)	113	406
Patients (*n*)	29,780	66,436
Daily number of dosage units administered	2.97 ± 0.88	3.02 ± 0.87
Hypnotics	0.74 ± 0.99	0.74 ± 0.98
Antihypertensives	0.66 ± 0.70	0.70 ± 0.71
Number of PIC per month	6.91 ± 3.13	7.03 ± 3.20
Number of beds, median [min–max]	87 [32–242]	80 [14–230]

*PIC* Presentation Identification Codes

**Table 2:** Comparison between intervention and control groups before and after the introduction of the booklet designed to aid physicians with prescribing in elderly nursing home residents

	Intervention group		Control group	
	Before (January 2011–April 2012)	After (May 2012–December 2014)	Before (January 2011–April 2012)	After (May 2012–December 2014)
Daily number of times drug dosage unit administered	3.00 (0.86)	2.95 (0.89)*	3.06 (0.85)	3.00 (0.88)*
Hypnotics	0.76 (1.00)	0.72 (0.97)*	0.77 (0.99)	0.72 (0.97)*
Antihypertensives	0.66 (0.71)	0.66 (0.69)	0.69 (0.71)	0.70 (0.71)
Number of PIC per month	7.07 (3.15)	6.81 (3.10)*	7.22 (3.23)	6.90 (3.18)*

*PIC* Presentation Identification Codes

*p < 0.001 for after versus before

### Difference-in-differences analysis and multivariable analysis

The multivariable analysis is presented in Table 3. Overall, there was significantly lower use of medications over the study period in the intervention group compared to the control group, with an RR of 0.98 (95% CI, 0.97 to 0.99). Similarly, considering both groups together, there was an overall reduction in medication use in the period after the implementation of the booklet, compared to the period before, with an RR of 0.98 (95% CI, 0.98 to 0.99). However, the interaction between the implementation of the booklet and the rate of use of medication was not significant in the difference-in-differences analysis, indicating that the reduction in medication use cannot be attributed to the implementation of booklet.

**Table 3:** Multivariable analysis of factors related to the average daily number of times when medications were administered

	Number of daily times drugs were administered	
	RR [95% CI]	*p* value
Intervention group (reference = control group)	0.98 [0.97–0.99]	0.02
After (reference = before)	0.98 [0.98–0.99]	< 0.001
Difference-in-differences—expressed as the ratio of RR	1.00 [0.99–1.02]	0.42
Number of beds	1.00 [1.00–1.00]	0.12
Public nursing home (reference = private for-profit)	0.99 [0.97–1.02]	0.65
Private not-for-profit nursing home (reference = private for-profit)	1.00 [0.98–1.02]	0.97

Analysis of the secondary assessment criteria revealed that the consumption of both antihypertensive drugs (Table 4) and hypnotics (Table 5) decreased significantly over time (RR 0.91 [0.89–0.92] and RR 0.94 [0.92–0.96], respectively). There was no difference between the intervention and control groups in the rate of use of either antihypertensives or hypnotics (Tables 4 and 5). The reduction in consumption over time could not be attributed to the intervention (*p* value for the difference-in-differences analysis 0.25 and 0.73 for antihypertensives and hypnotics, respectively). The results of the regression analyses of costs presented in Supplementary Table S2 and Supplementary Table S3 (see Additional File 1) confirm a decrease in medication use [Odds Ratio (OR) = 0.95; 95% Confidence Interval (CI) 0.95 to 0.97; *p* < 0001]. Similarly, we observed a significant association in multivariable analysis between public nursing homes and a lower cost of medications (OR = 0.68; 95% CI 0.47 to 0.99; *p* = 0.048).

**Table 4:** Multivariable analysis of factors related to the average daily number of times when antihypertensive drugs were administered

	Antihypertensive drugs	
	RR [95% CI]	*p* value
Intervention group (reference = control group)	0.96 [0.91–1.00]	0.07
After (reference = before)	0.91 [0.89–0.92]	< 0.001
Difference-in-differences—expressed as the ratio of RR	0.98 [0.94–1.02]	0.25
Number of beds	1.00 [1.00–1.00]	0.36
Public nursing home (reference = private for-profit)	0.94 [0.88–1.00]	0.05
Private not-for-profit nursing home (reference = private for-profit)	0.94 [0.90–0.99]	0.03

**Table 5:** Multivariable analysis of factors related to the average daily number of times when hypnotic drugs were administered

	Hypnotic drugs	
	RR [95% CI]	*p* value
Intervention group (reference = control group)	1.00 [0.94–1.05]	0.89
After (reference = before)	0.94 [0.92–0.96]	< 0.001
Difference-in-differences—expressed as the ratio of RR	1.00 [0.97–1.05]	0.73
Number of beds	1.00 [1.00–1.00]	0.67
Public nursing home (reference = private for-profit)	1.04 [0.98–1.12]	0.21
Private not-for-profit nursing home (reference = private for-profit)	0.98 [0.93–1.04]	0.49

## Discussion

Our study shows that there was a decrease in medication use in nursing homes in terms of daily doses administered in both the intervention group and the control group. Our results also indicate that the average monthly number of PIC decreased significantly between both periods. The difference-in-differences analysis indicates that these effects could not be attributed solely to the introduction of the booklet. The failure to show any effect of the booklet on prescribing practices in this study may be explained by the simultaneous existence of a national policy promoting quality of care through continuing medical education, which may have affected both groups in a similar manner. However, the national policy highlighted the need for improved coordination and better monitoring of prescriptions in nursing home residents, without providing any concrete measure for implementation in practice in nursing homes. Nursing homes are also free to promote the appropriate use of medication through various local programs led by pharmacists or physicians, who often participate in continuing medical education.

The aim of our study was to observe specific trends in drug prescriptions in nursing homes, and to assess the impact of the implementation of a designated educational booklet on the prescription of medications. Our study included a high number of facilities and measured drug prescriptions with an automated data collection process.

Our observation of a general decrease in drug consumption over the study period is in agreement with the data in the literature [40, 41], although our analysis precludes any conclusion that the booklet was the driving force behind this reduction. There is a growing body of evidence in the literature in favor of the efficacy of educational approaches in reducing polypharmacy [42]. A study by Blochet et al. [34] using a similar electronic pill dispensing system observed an overall decrease in the number of tablets taken per resident per day from 2011 to 2013 in a sample of 338 nursing homes in France. A recent study of prescriptions in 1890 residents in nursing homes in France using national health insurance data reported that 42.9% had polypharmacy (defined as 5 to 9 drugs per day) and 46.7% of the study sample received at least one potentially inappropriate medication [43]. In a study using the same pill-dispenser database as in our analysis, these same authors also reported a significantly lower risk of excessive polypharmacy in private for-profit nursing homes, with an OR (reference group: public nursing homes) of 0.81 (95% CI, 0.68 to 0.98, *p* = 0.026) [9]. Our intervention group comprised nursing homes that were all private for-profit establishments, although we did not observe any effect of nursing home status on overall rate of drug use. The cost of medications appeared to be lower in public nursing homes, likely because many public nursing homes are affiliated to a centralized purchasing department, enabling group procurement and, consequently, lower prices. Deprescribing potentially harmful or inappropriate medications in polymorbid older adults is an attractive option that has been shown to be useful in reducing the risks associated with polypharmacy [44, 45]. In this regard, our overall finding of a reduction in prescriptions may be a step in the right direction towards improved quality of care. However, reviews of the literature and meta-analyses evaluating the efficacy of interventions to reduce polypharmacy have yielded contradictory results, with a relatively limited impact on mortality and hospitalizations [46]. In addition, we cannot rule out that other factors may have contributed to this overall decline in drug use over time, such as a downward change in the care burden of the nursing home residents.

### Study strengths and limitations

The strengths of this study include the fact that this is a nationwide study in a large number of nursing homes, aimed at measuring the potential effect of the implementation of a booklet targeting appropriate drug prescriptions. The study sample is very large and likely representative of the general population of nursing home residents in France. Furthermore, the long (4-year) study period was sufficient to capture lasting changes in prescription trends, and the appropriate statistical approach was used to control for potential confounders. Our study also has some limitations. First, it was not a randomized study and there were differences between nursing homes in the intervention group and the control group. In addition, we cannot exclude the possibility that there may have been changes in the demographics and/or number of prescribers over the course of the study period. However, these differences were accounted for in the statistical analysis by adjusting for confounders. Moreover, because of its size and representativeness, the control group adequately reflected trends in medication use for nursing homes. Indeed, since all nursing homes in the study used the pill dispenser, which has been proven to be reliable, and since the prescriptions are written by GPs who are not employees of the nursing home, then we believe that the use of the electronic pill dispensing system would have no influence on prescribing practices.

Second, our measure of medication use may not cover the entire range of drug prescriptions, as we only had data derived from the residents’ pill dispensers, nor does it distinguish between drugs prescribed for acute versus chronic diseases. This does not, however, limit the interpretation of the results, since the general decrease in drug consumption likely reflects a decrease in prescriptions by physicians. Third, staff-related confounders (e.g., increased diligence in applying the intervention among some staff members) could not be measured or accounted for the analysis. Fourth, we were unable to monitor the uptake or use of the booklet by physicians; therefore, it is possible that the value of the booklet was underestimated in our study. In addition, we had no information regarding similar information campaigns that may have been undertaken in the control group during the study period. Despite the intensive communication campaigns about the booklet in the nursing homes of the intervention group, there is no guarantee that the prescribers actually followed the booklet’s guidance, and its use was not enforced or otherwise mandated. Moreover, the effects of a change in behavior are not immediately visible, although our 4-year study period should have been sufficient to reveal any effect of the intervention. Finally, our approach was limited to pill dispensers, and we could not compare our findings to clinical data, which might have allowed us to assess the impact on residents in terms of adverse effects.

## Conclusion

Our study observed a sizeable reduction in drug consumption in both the intervention and the control groups over the study period, but failed to show any significant impact of the booklet on prescribing practices in the intervention group. Our results show that using data routinely collected by electronic pill dispensers, drug prescriptions in nursing homes can be used as quality indicators for the evaluation of drug safety, and for monitoring purposes. These data could be helpful in identifying sources of differences in prescribing practices between nursing homes. Future research in this area could investigate the seemingly consequential differences in costs, as well as the impact of quality indicators for prescription on drug prescription practices.

## Electronic supplementary material

Below is the link to the electronic supplementary material.

Supplementary file1 (DOCX 21 kb)

## Data Availability

The datasets used and/or analyzed during the current study are available from the corresponding author on reasonable request.
