# Supplementary material for: No impact of a prescription booklet on medication consumption in nursing home residents from 2011 to 2014: a controlled before–after study
Source: Aging Clin Exp Res. 2020 Aug 3;33(6):1599–607. doi: 10.1007/s40520-020-01670-5 (PMC8203501; doi:10.1007/s40520-020-01670-5)
Supplement: Supplementary file 1 — Supplementary file1 (DOCX 21 kb) [file 40520_2020_1670_MOESM1_ESM.docx]

**Supplementary Material**

**No Impact of a Prescription Booklet on Medication Consumption In Nursing Home Residents from 2011 to 2014: A Controlled Before-After Study**

Stéphane Sanchez, MD, MPH ^1,2^, Cécile Payet, MPH ^3^, Marie Herr, PharmD, PhD ^4^, Fiona Ecarnot, PhD ^5,6^*, Caroline Blochet, PharmD ^7^, Didier Armaingaud, MD ^2^, Jan Chrusciel, MD ^1^, Jean-Luc Novella, MD, PhD ^8,9^, Rachid Mahmoudi, MD, PhD^8,9^.

Supplementary tables

- Table S1: Cost of medication

- Table S2: Multivariable analysis for the number of medications (i.e. different presentation identifier codes) per month per resident

- Table S3: Multivariable analysis for cost per entity per month.

**Supplementary Table S1: Cost of medication**

| **Nursing home** | **Intervention group** | **Control group** |
| --- | --- | --- |
| Total cost of medication | 8,447,860 € | 6,323,764 € |
| Cost of hypnotics | 219,160 € | 165,386 € |
| Cost of antihypertensives | 557,820 € | 435,573 € |
| Cost of medication per patient (2011 to 2014) | 283.68 € | 72.56 € |
| Cost of hypnotics | 7.36 € | 1.89 € |
| Cost of antihypertensives | 18.73 € | 4.92 € |

**Supplementary Table S2: Multivariable analysis for the number of medications (i.e. different presentation identifier codes) per month per resident**

| **Explanatory variables** | **RR [95% CI]** | ***P*-value** |
| --- | --- | --- |
| Intervention group (reference = control group) | 0.98 [0.95-1.01] | 0.225 |
| After (reference = before) | **0.95 [0.95-0.97]** | **< 0.001** |
| Difference-in-differences (expressed as the ratio of RR) | 1.00 [0.99-1.02] | 0.446 |
| Number of beds | 1.00 [1.00-1.00] | 0.204 |
| Public entity (reference = private for-profit) | 0.99 [0.94-1.04] | 0.863 |
| Private not-for-profit (reference = private for-profit)^a^ | 1.00 [0.96-1.02] | 0.868 |

^a^ Public nursing homes are not for profit

Abbreviations: CI = Confidence Intervals; RR = Relative Risks

**Supplementary Table S3: Multivariable analysis for cost per entity per month**

| **Explanatory variables** | **RR [95% CI]** | ***P*-value** |
| --- | --- | --- |
| Intervention group (reference = control group) | 1.16 [0.61-2.21] | 0.637 |
| After (reference = before) | 0.85 [0.66-1.11] | 0.251 |
| Difference-in-differences (expressed as the ratio of RR) | 0.79 [0.48-1.30] | 0.369 |
| Number of beds | 1.00 [0.99-1.00] | 0.940 |
| Public entity (reference = private for-profit) | **0.68 [0.47-0.99]** | **0.048** |
| Private not-for-profit (reference = private for-profit)^a^ | 0.76 [0.51-1.14] | 0.195 |

^a^ Public nursing homes are not for profit

Abbreviations: CI = Confidence Intervals; RR = Relative Risks
